# Effect-site concentration of remifentanil for smooth emergence from sevoflurane anesthesia in patients undergoing endovascular neurointervention

**DOI:** 10.1371/journal.pone.0218074

**Published:** 2019-06-11

**Authors:** Ji-Hye Kwon, Young Hee Shin, Nam-Su Gil, Jungchan Park, Yoon Joo Chung, Tae Soo Hahm, Ji Seon Jeong

**Affiliations:** Department of Anesthesiology and Pain Medicine, Samsung Medical Center, Sungkyunkwan University School of Medicine, Seoul, Korea; University of Mississippi Medical Center, UNITED STATES

## Abstract

During emergence from general anesthesia, coughing caused by the endotracheal tube frequently occurs and is associated with various adverse complications. In patients undergoing endovascular neurointervention, achieving smooth emergence from general anesthesia without coughing is emphasized since coughing is associated with intracranial hypertension. Therefore, the up-and-down method was introduced to determine the effective effect-site concentration (Ce) of remifentanil to prevent coughing in 50% and 95% (EC_50_ and EC_95_) of patients during emergence from sevoflurane anesthesia for endovascular neurointervention. A total of 43 participants, American Society of Anesthesiologists class I or II participants, aged from 20 to 70 years who were undergoing endovascular neurointervention through transfemoral catheter for cerebrovascular disease were enrolled. Using the up-and-down method with isotonic regression, the EC_50_ and EC_95_ of remifentanil to prevent coughing during emergence from sevoflurane anesthesia were determined. We also investigated differences of hemodynamic and recovery profiles between the cough suppression group and the cough group. In total, 38 of 43 patients were included for estimation of EC_50_ and EC_95_. The EC_50_ and EC_95_ of remifentanil to prevent coughing were 1.42 ng/mL (95% confidence interval [CI], 1.28–1.56 ng/mL) and 1.70 ng/mL (95% CI, 1.67–2.60 ng/mL), respectively. There was comparable emergence and recovery data between the cough suppression group (n = 22) and the cough group (n = 16). However, the Ce of remifentanil and total dose of remifentanil were significantly higher in the cough suppression group (P = 0.002 and P = 0.004, respectively). Target-controlled infusion of remifentanil at 1.70 ng/mL could effectively prevent extubation-related coughing in 95% of neurointervention patients, which could ensure smooth emergence.

## Introduction

While emerging from general anesthesia, coughing induced by the endotracheal tube (ETT) frequently occurs and is associated with various adverse complications including intracranial hypertension [1], increased intraocular pressure [2], hypertension, and tachycardia [3]. There have been efforts to prevent coughing while emerging from anesthesia, and prevention is emphasized in specific clinical situations.

Opioids are a commonly used therapeutic option to prevent and resolve coughing in patients with respiratory disease as well as with airway irritation [4,5]. Previous studies demonstrated the antitussive effect of remifentanil for emergence from anesthesia under various settings and reported effective and safe suppression of coughing by target-controlled infusion (TCI) of remifentanil [6–8]. Remifentanil is known as a short-acting synthetic opioid analgesic and has the antitussive effect affecting brainstem μ-opioid receptors [5]. However, various factors including patient age, sex, procedures, and anesthetic agents affect the effect-site concentration (Ce) of remifentanil [4,6,9–11]. Therefore, remifentanil administration to prevent coughing during emergence should be customized to the specific settings [4].

In patients under endovascular neurointervention due to neurological disease, it is crucial to obtain gentle emergence from anesthesia without coughing since cough caused by ETT is associated with intracranial hypertension [1]. Hemodynamic instability by stimulation can increase the incidence of intracranial bleeding or rupture of aneurysms. Therefore, patients undergoing neurointervention should be gently recovered without coughing and hemodynamic instability. Nonetheless, no specific reports on smooth emergence in patients going through endovascular neurointervention with sevoflurane anesthesia have been found. In particular, neurointervention is characterized by a procedure with minimal pain that requires a smaller dose of remifentanil for smooth emergence than other painful invasive surgeries [12].

The purpose of this study was to find the effective Ce of remifentanil to prevent coughing in 50% and 95% (EC_50_ and EC_95_) of patients during emergence from sevoflurane anesthesia after endovascular neurointervention.

## Methods

This prospective study was approved by the Institutional Review Board of Samsung Medical Center (IRB No. SMC 2017-10-061-001) and registered at the Clinical Research Information Service (CRIS, https://cris.nih.go.kr) under the identification number KCT0002691 (date of registration: 12/12/2017). This investigation was conducted at a tertiary medical center, Samsung Medical Center, in Seoul, Korea, between February 2018 and May 2018.

### Patients

A total of 43 participants with an American Society of Anesthesiologists (ASA) class Ⅰ or Ⅱ, aged from 20 to 70 years who were undergoing neurointervention because of cerebrovascular disease were enrolled. After sufficient information regarding participation in this study was provided to patients, written informed consent was obtained. Exclusion criteria were patients who had symptoms or signs of increased intracranial pressure, malignant hypertension, preoperative administration of an antitussive agent, allergic history with use of remifentanil or contraindication to remifentanil, gastroesophageal reflux, arrythmia or congestive heart failure (New York heart association heart failure grade 3,4), severe obesity (body mass index > 35 kg/m^2^), severe obstructive sleep apnea, anticipated difficult airway, acute or chronic respiratory disease (chronic obstructive lung disease, bronchial asthma, upper respiratory infection within 2 weeks, etc.), or patients who require ventilator care after neurointervention.

### Anesthetic protocol

Patients were not premedicated and were evaluated before neurointervention. Patients were monitored with standard monitors including oxygen saturation (SpO_2_), electrocardiography, noninvasive blood pressure, neuromuscular monitoring, and bispectral index (BIS). Anesthesia was induced with 5 mg/kg of thiopental sodium and 4 vol% of sevoflurane and remifentanil effect-site TCI of 2 ng/mL. For effect-site TCI of remifentanil, a commercial TCI pump (Orchestra Base Primea, Fresinus Vial, France) was used, and the pharmacokinetic set used to calculate target Ce was the Minto model [13]. After loss of consciousness, rocuronium 0.8 mg/kg was given intravenously, and manual ventilation with 100% O_2_ was performed. Tracheal intubation was conducted with a 7.0 mm (internal diameter [I.D.]) ETT for women and 8.0 mm for men after confirming muscle relaxation with train-of-four (TOF) counts of 0, and cuff pressure was adjusted between 20 and 25 mm H_2_O with a hand pressure gauge. After intubation, supplementary monitors such as those for end-tidal carbon dioxide (EtCO_2_), oroesophageal temperature, and invasive arterial blood pressure through a radial artery catheter were applied. Anesthesia was continued with sevoflurane and effect-site TCI of remifentanil to control a BIS target level of hypnosis of 40 to 60 and to control blood pressure and heart rate (HR) within 20% of baseline values. Hypotension (baseline mean arterial pressure [MAP] < 20%) was treated with 5 mg ephedrine, and bradycardia (baseline HR < 20%) was treated with 0.5 mg atropine. Mechanical ventilation was maintained with a tidal volume of 8 mL/kg, and ventilator frequency was controlled to maintain EtCO_2_ at 35 to 40 mmHg. Patient body temperature was controlled at a target value of 36.5ºC.

About 15 minutes before procedure completion, sevoflurane concentration was controlled to maintain a BIS value of 60, and the Ce of remifentanil was set to a predetermined concentration. After completion of the procedure, oropharyngeal suction was gently performed, and 2–4 mg/kg of sugammadex determined by TOF value was injected to reverse neuromuscular block. After confirming > 1.0 TOF ratio [14], sevoflurane was discontinued. In addition, a predetermined concentration of remifentanil was maintained until extubation. An anesthesiologist assisted ventilation to maintain an EtCO_2_ of 40 to 50 mmHg. The patient received a verbal request continuously to open their eyes without any other stimulus. After eye opening, patients were asked to breathe deeply. When adequate spontaneous respiration was confirmed, ETT was removed with cuff deflated. Remifentanil was discontinued after extubation, and oxygen was supplied via facial mask for 3 minutes. When the patient reached a stable state, she/he was moved to the postanesthetic care unit (PACU). If recovery time exceeded 20 minutes after cessation of sevoflurane, the patient was excluded from the study as it was considered delayed emergence.

### Determining remifentanil dose and blinding

Remifentanil was administered to prevent coughing during emergence using Dixon’s up-and-down method [15]. The initial Ce of remifentanil was 2.0 ng/ml for the first patient, based on the EC_95_ previously reported [6,7]. We defined cough as a strong and sudden contraction of the abdomen muscles during emergence. If the patient was determined to have a cough during emergence, it was considered as failure, and the next patient’s Ce of remifentanil was increased by 0.2 ng/ml (10% of the initial Ce of remifentanil). Conversely, if the patient showed no cough during emergence, it was considered as success, and the next patient’s Ce of remifentanil was decreased by 0.2 ng/mL. In previous studies, dose for a step up or down in the up and down process were various from 0.4 to 0.5 ng/mL in various kinds of surgeries [6,7,16]. Because neurointervention is characterized by a procedure with minimal pain, we expected relatively less effect-site concentration compared to other studies. Therefore, we decided to set the dose 0.2 ng/ml as a step up or down to find the fine concentration.

Two anesthesiologists participated in the procedure. The first anesthesiologist titrated the remifentanil dose using a TCI pump and recorded remifentanil dose, end-tidal sevoflurane concentration (EtSevo), EtCO_2_, and other hemodynamic parameters during the procedure and emergence. The second anesthesiologist who was blinded to the patients’ Ce of remifentanil assessed the occurrence of cough and other complications during emergence and recorded the outcome. Recovery data including Richmond Agitation and Sedation Scale (RASS), modified Aldrete score, numeric rating scale (NRS), any complication, and duration of PACU stay were measured by the other investigator.

The primary outcome of this study was to estimate the EC_50_ and EC_95_ of remifentanil to prevent coughing during recovery from sevoflurane anesthesia after endovascular neurointervention. We also investigated the differences in intraoperative, emergence, and recovery data between the cough group and cough suppression group.

### Statistical analysis

Based on similar studies involving binary outcomes, we estimated that a minimum of 10 dependent negative-positive up-and-down deflections were required to calculate the EC_50_ and EC_95_ [7,17,18]. The data were analysed with isotonic regression to calculate the Ce of remifentanil required to prevent cough during emergence in 50% (EC_50_) and 95% (EC_95_) of subjects. An adjusted response probability was calculated by the pooled adjacent-violators algorithm (PAVA) [19,20], and the confidence interval (CI) for isotonic regression used the recursive algorithm by Morris for ordered-binomial point estimates [21,22]. Isotonic regression is a well-described variant of restricted least squares regression that constrains the point estimates to be either monotonic increasing (never decreasing) or monotonic decreasing (never increasing). The isotonic regression estimator method has less bias and greater precision than the other up-and-down method estimators for calculating the EC_50_ and EC_95_ [15]. Although previous studies used bootstrap for CI, Iassons and Ostrovnaya argued that the bootstrap method is inadequate for use in CI of isotonic regression [22,23]. Pearson correlation was used to evaluate correlations between Ce of remifentanil (total remifentanil dose) and time to extubation and duration of PACU stay. Continuous variables, except for estimated EC_50_ and EC_95_, are presented as mean ± SD or median (interquartile range [IQR]) as appropriate, and categorical variables are presented as number (%). Normality of all continuous variables was assessed by Shapiro-Wilk test. Comparison of intraoperative, emergence, and recovery data between the cough suppression and cough group was performed using t-test, Wilcoxon rank sum test, chi-square test, and Fisher’s exact test.

P-value < 0.05 was considered statistically significant. Statistical analysis was executed using SPSS version 22 (SPSS Inc., Chicago, IL, USA) and R statistical software version 3.3.2 (Vienna, Austria; http://www.R-project.org/).

## Results

**Fig 1:**
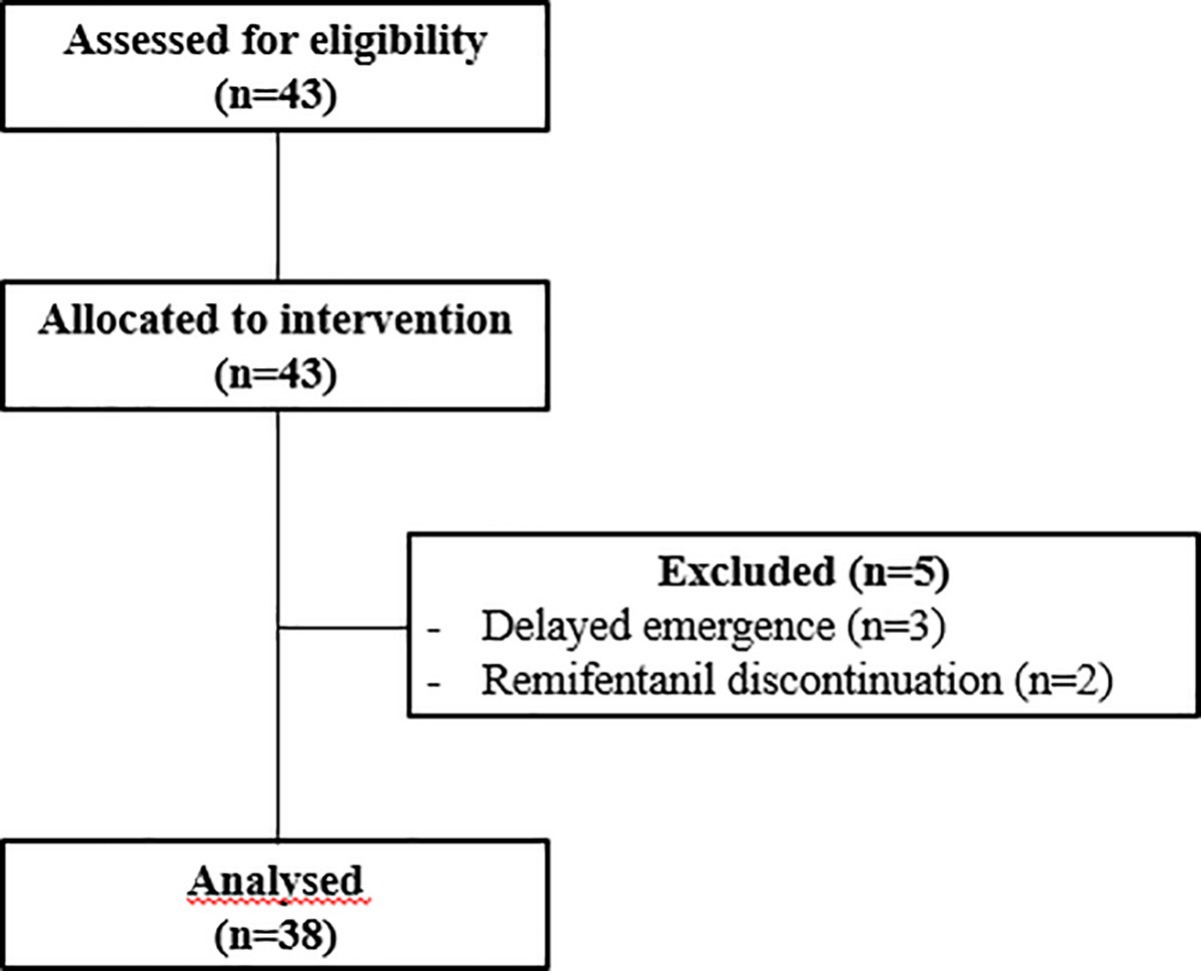
Flow diagram.

**Fig 2:**
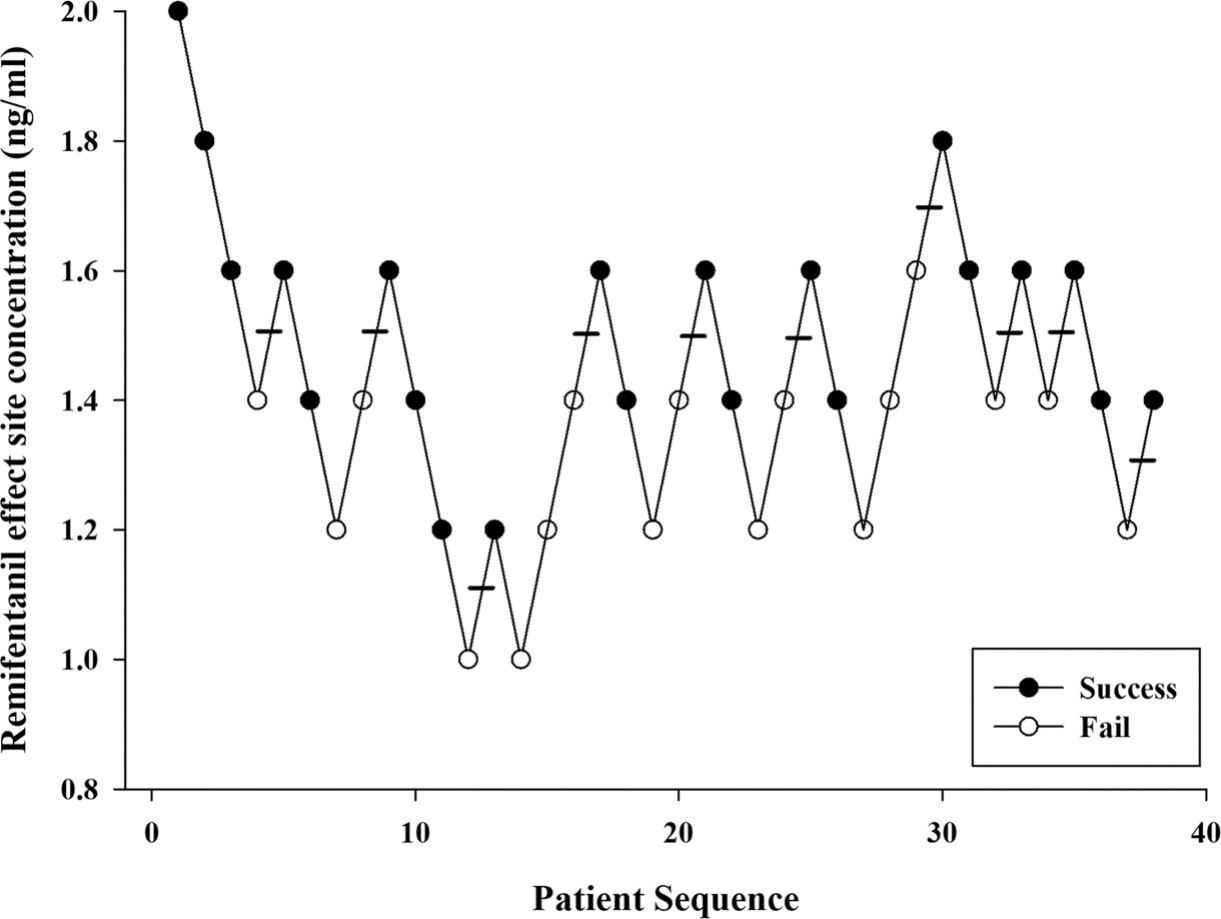
The up-and-down sequence of remifentanil to prevent cough during the emergence period in endovascular neurointervention.

**Fig 3:**
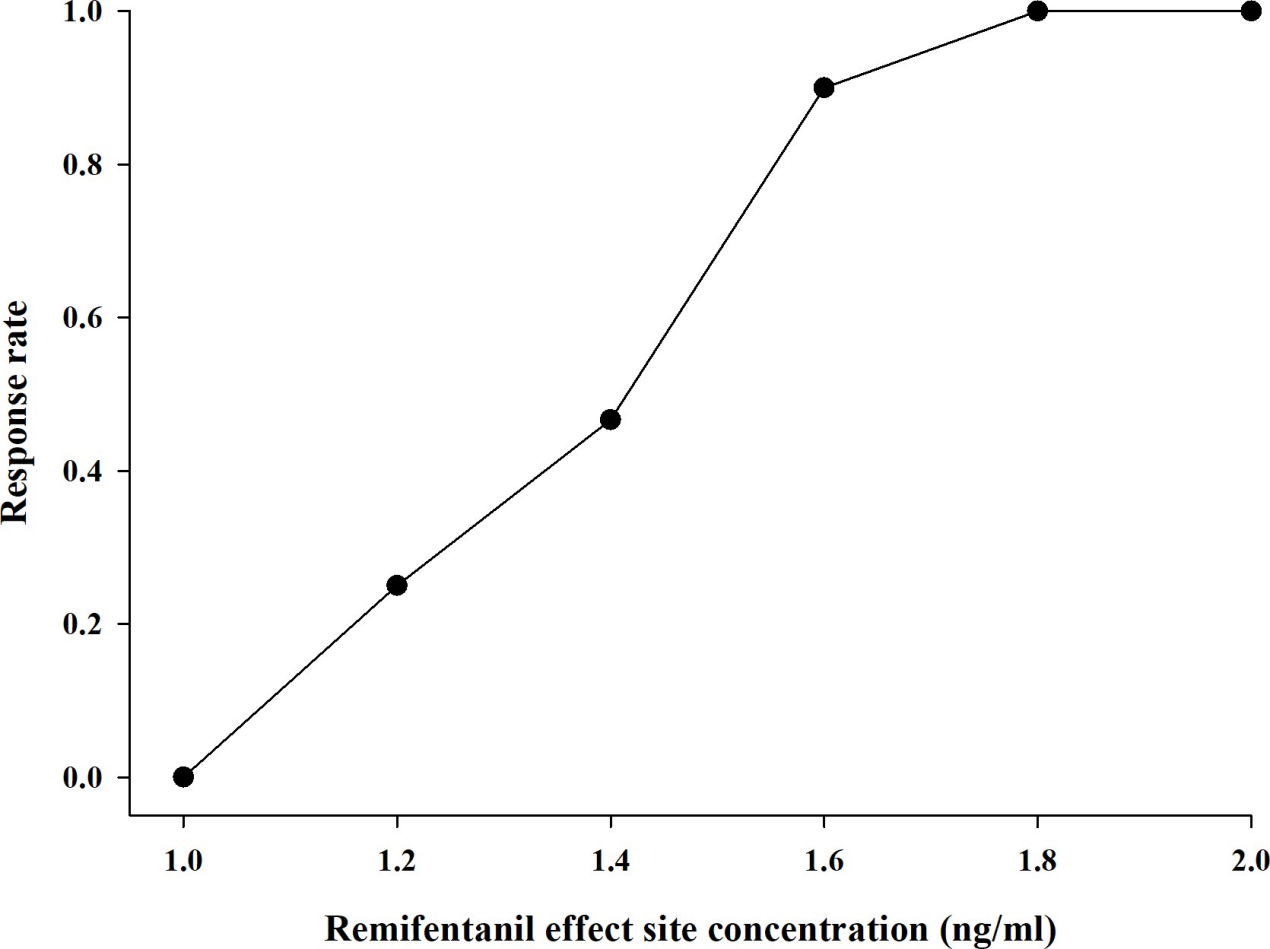
Pooled adjacent violators algorithm (PAVA) response rate.

A total of 43 patients were enrolled using Dixon up-and-down methods. Five patients were excluded for delayed emergence and remifentanil discontinuation due to low blood pressure. A total of 38 patients were included for estimation of EC_50_ and EC_95_ (Fig 1). Demographic characteristics are reported in Table 1. Figs 2 and 3 demonstrate the up-and-down results in consecutive patients and PAVA response rate, respectively. The EC_50_ and EC_95_ of remifentanil as estimated by the isotonic regression model were 1.42 ng/mL (95% CI, 1.28–1.56 ng/mL) and 1.70 ng/mL (95% CI, 1.67–2.60 ng/mL), respectively. The correlation coefficients between Ce of remifentanil and time to extubation and duration of PACU stay were -0.011 and 0.473, respectively (P = 0.95 and P = 0.003).

**Table 1:** Demographic characteristics.

	Cough suppression group (n = 22)	Cough group (n = 16)
Age (years)	56.41 ± 9.63	55.81 ± 7.88
Height (cm)	162.21 ± 10.12	161.23 ± 7.37
Weight (kg)	66.66 ± 12.23	65.34 ± 11.39
Sex		
Female	12	10
Male	10	6
ASA (Ⅰ / Ⅱ)	1/21	0/16
Disease type		
Aneurysm	14	9
AVM	7	7
ICA stenosis	1	0

Data are presented as mean ± SD or number.

ASA: American Society of Anesthesiologists; AVM: arteriovenous malformation; ICA: internal carotid artery.

Intraoperative and emergence data between the cough suppression group (n = 22) and cough group (n = 16) are reported in Table 2. The Ce of remifentanil and total dose of remifentanil were significantly higher in the cough suppression group (P = 0.002 and P = 0.004, respectively). The recovery data in the PACU between the two groups were comparable (Table 3). In both groups, pain control was not needed. Postoperative nausea and vomiting (PONV) in PACU was 7/22 (32%) in the cough suppression group and 2/16 (13%) in the cough group (P = 0.25).

**Table 2:** Comparison of intraoperative and emergence data between cough suppression group and cough group.

	Cough suppression group (n = 22)	Cough group (n = 16)	P
**Intraoperative data**			
Duration of procedure (min)	100.0 (90.0–108.0)	95.0 (79.0–112.5)	0.76
Duration of anesthesia (min)	145.0 (135.0–166.0)	136.5 (120.5–160.0)	0.23
Ce of remifentanil (ng/mL)	1.6 (1.4–1.6)	1.4 (1.2–1.4)	**0.002**
Total dose of remifentanil (mg)	0.5 (0.45–0.6)	0.4 (0.35–0.5)	**0.004**
**Emergence data**			
Time to eye opening (min)	11.9 ± 4.9	10.9 ± 4.2	0.53
Time to extubation (min)	12.6 ± 4.6	11.6 ± 3.9	0.45
MAP (mmHg)			
At the end of procedure	78.9 ± 13.8	81.4 ± 15.9	0.62
Before extubation	101.7 ± 16.9	107.3 ± 20.2	0.36
After extubation	106.9 ± 13.5	114.3 ± 17.7	0.15
3 min after extubation	98.3 ± 23.9	105.3 ± 13.5	0.30
HR (beats / min)			
At the end of procedure	59.2 ± 11.3	60.1 ± 8.6	0.79
Before extubation	75.3 ± 11.9	82.4 ± 7.4	**0.04**
After extubation	82.9 ± 9.9	89.2 ± 10.7	0.07
3 min after extubation	81.5 ± 9.5	86.6 ± 10.8	0.13
RR (breaths / min)			
At the end of procedure	10 (9–10)	10 (10–11)	0.11
Before extubation	10 (10–12)	10 (10–10)	0.78
After extubation	10 (10–12)	10 (10–15)	0.26
3 min after extubation	10 (10–12)	10 (10–12)	0.85
EtCO_2_ (mmHg)			
At the end of procedure	34.1 ± 4.7	34.1 ± 3.0	0.96
Before extubation	41.8 ± 7.4	43.3 ± 6.6	0.53
After extubation	40.9 ± 7.2	42.2 ± 6.9	0.59
3 min after extubation	40.1 ± 8.4	40.3 ± 7.2	0.92
EtSevo (vol%)			
At the end of procedure	0.74 ± 0.19	0.79 ± 0.16	0.44
Before extubation	0.17 ± 0.05	0.19 ± 0.06	0.11
After extubation	0.16 ± 0.04	0.18 ± 0.04	0.13
3 min after extubation	0.12 ± 0.04	0.12 ± 0.04	0.57
BIS value			
At the end of procedure	59.8 ± 1.8	60.3 ± 2.2	0.42
Before extubation	84.3 ± 6.5	80.5 ± 8.6	0.13
After extubation	85.9 ± 4.7	83.0 ± 6.3	0.12
3 min after extubation	86.1 ± 4.6	83.0 ± 4.9	0.05

Data are presented as mean ± SD or median (range).

Ce: effect-site concentration; MAP: mean arterial pressure; HR: heart rate; RR: respiratory rate; EtCO_2:_ end-tidal carbon dioxide concentration; EtSevo: end-tidal sevoflurane concentration; BIS: bispectral index.

**Table 3:** Comparison of recovery data in PACU between cough suppression group and cough group.

	Cough suppression group (n = 22)	Cough group (n = 16)	P
Duration of PACU stay (min)	50.7 ± 9.5	44.9 ± 9.8	0.08
RR (breaths / min)			
Admission	15 (14–18)	15 (12–16)	0.18
10 min after PACU admission	18 (13–19)	14 (13–20)	0.44
20 min after PACU admission	15.0 (14–17)	15 (14–19)	0.68
Discharge	16.0 (13–18)	16.0 (13–20)	0.42
RASS			
Admission	2 (1–2)	2 (2–2)	0.22
10 min after PACU admission	1 (1–2)	1 (1–2)	0.90
20 min after PACU admission	1 (1–1)	1 (1–2)	0.51
Discharge	1 (1–1)	1 (1–1)	0.83
Modified Aldrete score			
Admission	9 (9–10)	9 (9–9)	0.65
10 min after PACU admission	10 (9–10)	9 (9–10)	0.51
20 min after PACU admission	10 (10–10)	10 (9–10)	0.22
Discharge	10 (10–10)	10 (10–10)	0.65
Complication			
Pain (NRS > 3)	0 (0)	0 (0)	-
PONV	7 (32)	2 (13)	0.25

Data are presented as mean ± SD, median (range) or number (%).

PACU: postanesthetic care unit; RR: respiration rate; RASS: Richmond Agitation and Sedation Scale; NRS: numeric rating scale; PONV: postoperative nausea vomiting.

One patient suffered a desaturation event during the emergence period but recovered spontaneously without any respiratory complications. Significant respiratory complications did not occur in any patients.

## Discussion

The main finding of our study is that the EC_50_ and EC_95_ of remifentanil to prevent coughing during emergence from sevoflurane anesthesia are 1.42 ng/mL (95% CI, 1.28–1.56 ng/mL) and 1.70 ng/mL (95% CI, 1.67–2.60 ng/mL), respectively, in patients undergoing endovascular neurointervention through transfemoral catheter. When the Ce of remifentanil increased, duration of PACU stay was prolonged, but the time of extubation was not statistically different. Among the patients included in this study, 22 had no coughing (cough suppression group) and 16 experienced coughing (cough group). There were significant differences in Ce of remifentanil and total amount of remifentanil, with higher values in the cough suppression group.

In a previous study, coughing caused by ETT during emergence was effectively suppressed by the use of remifentanil [6,7,16,24]. In the studies, except for pediatric patients, remifentanil to prevent coughing was calculated using TCI, and the average estimated EC95 of remifentanil in each case was > 2.0 ng/ml: 2.51 ng/mL (95% CI, 2.28–2.57 ng/mL) in patients undergoing transsphenoidal surgery, 2.27 ng/mL (95% CI, 1.95–2.26 ng/mL) in patients intubated with a double lumen ETT, and 2.14 ng/mL (95% CI, 1.89–3.57 ng/mL) in patients undergoing thyroid surgery [6,7,16]. In our study, the EC95 of remifentanil to prevent coughing was 1.70. Although the upper limit of our confidence interval exceeded 2 (95% CI, 1.67–2.60 ng/mL), thus overlapping estimates from other studies, it is difficult to directly compare CIs as they are calculated in different ways. Notably, no patients in our study required rescue analgesics during the recovery period, and the NRS did not exceed 3. Therefore, the EC95 of remifentanil we report, for procedure with minimal pain (as compared to those reported for the above studies), supports a relatively lower concentration than that reported in previous studies.

The use of opioids during the intraoperative period increased the incidence of PONV. A remarkable dose-response relationship between opioid use and PONV has been reported in several studies [25,26]. In our study, Ce of remifentanil and total dose of remifentanil were significantly higher in the cough suppression group compared to the cough group, which means that a relatively larger dose of remifentanil was administered in the cough suppression group and led to successful cough suppression.

Remifentanil is characterized by rapid onset, peak effect, and ultra-short duration of action. In contrast to these characteristics, Chang et al. demonstrated that patients with remifentanil infusion at Ce 2 ng/mL showed delayed awakening and low level of consciousness in the PACU compared to patients with remifentanil infusion at Ce 1 ng/mL or Ce 1.5 ng/mL [27]. Also, there was a tendency for dose-dependent increase in duration of PACU stay [27]. In our study, the Ce of remifentanil showed significant correlation with duration of PACU stay, but not with time to extubation. Therefore, a higher dose of remifentanil intraoperatively will prolong the duration of the PACU stay.

As the depth of anesthesia declined after cessation of anesthetic agents, the chance of coughing increases because of the constant stimulus by ETT [10]. Various methods to prevent coughing during emergence have been introduced including the administration of dexmedotomidine [28], deep extubation [29], intravenous or topical lidocaine [2,30], lidocaine applied inside the tracheal tube cuff [31], and administration of remifentanil [6,7,16]. Among these, recent studies demonstrated that a TCI of remifentanil using a predicted Ce can prevent coughing during emergence safely and effectively [6,7,16]. As the antitussive effect of opioids is central, regulated by brainstem opioid receptors [5,32], administration of remifentanil by TCI enables maintenance of a steady and reliable Ce compared to intravenous bolus administration. Also, administration of remifentanil during anesthetic emergence was demonstrated to have a remarkable antitussive effect compared to intravenous injection of lidocaine [33]. However, various factors including patient age, sex, procedures, and anesthetic agents affect the dose of remifentanil to prevent coughing [4,6,9–11], so remifentanil administration to prevent coughing during emergence should be customized to the specific settings [4].

Endovascular neurointervention through transfemoral catheter has been a preferred technique for diagnosis and treatment of neurovascular abnormalities such as arteriovenous malformations and aneurysms by providing images of blood vessels in and around the brain [12,34]. For some applications, endovascular neurointervention may provide better images than less invasive images such as computed tomography angiography and magnetic resonance angiography. In addition, endovascular neurointervention enables operators to treat disease immediately, based on its findings. For the interventional procedure for neurovascular abnormalities, metal coils or stents may be introduced through the femoral catheter already in place and maneuvered to the site of aneurysm [12]. In our study, patients had neurovascular diseases including cerebral aneurysm, arterio-venous malformation, and internal carotid artery stenosis. In patients with neurovascular disease, coughing during emergence was associated with intracranial hypertension and increased intracranial pressure after neurointervention. This hemodynamic stimulation increases the risk of intracranial bleeding and rupture of aneurysms [1]. Therefore, it is crucial to obtain smooth emergence from anesthesia without coughing in the setting of endovascular neurointervention. Although, we failed to find statistically different hemodynamic parameters except HR before extubation, our results showed higher trend of MAP and HR in cough group.

We used sugammadex for reverse of neuromuscular blocking agent (NMBA) and proceeded to the emergence process with a > 1.0 TOF ratio to prevent residual blocks. However, in another study, neostigmine was used for NMBA reversal, and a > 0.9 TOF ratio was determined to prevent residual blocks [6]. Using sugammadex with > 1.0 TOF ratio before emergence, the residual effect of NMBA was nearly excluded when evaluating the effect of remifentanil to prevent coughing. However, the use of sugammadex for reversal of NMBA results in more coughing during emergence than use of neostigmine, and gradual recovery of NMBA is important to prevent coughing [35]. Therefore, the depth of anesthesia must gradually decrease so that coughing caused by sudden recovery of NMBA after sugammadex administration could be minimized. In addition, Doi et al. reported that sevoflurane was the least irritative agent of volatile anesthetics [36]. Therefore, in our study, the effect of other anesthetic agent such as NMBA and sevoflurane that could cause cough can be considered minimized.

Our study has several limitations. First, the incidence of cough during emergence from general anesthesia has various results depending on the type of anesthetic agent including propofol and sevoflurane [4,10]. Our study investigated the EC_50_ and EC_95_ of remifentanil to prevent coughing during emergence in sevoflurane anesthesia; however, depending on the type of anesthetic agent such as volatile anesthetics or propofol, the activity of airway receptors may be affected, and there may be difference in remifentanil requirements [37]. Therefore, our findings cannot be extrapolated to other anesthetic methods. Second, the demographic characteristics including sex and age of the enrolled patients were not homogeneous. In children, remifentanil showed a rapid elimination similar to that in adults [38]. The elderly tend to have a smaller volume of distribution and a slightly lower clearance and dose of remifentanil should be decreased with increasing age [39]. The esterase-based metabolism of remifentanil makes its pharmacokinetics independent of end-organ failure including hepatic and renal failure [39]. Several studies revealed that the requirements of opioids to prevent coughing vary depending on sex; however, our study included both men and women [9,11]. The pharmacokinetic of remifentanil is no influence of gender [13], however sex affects the analgesic effects of opioids and the requirements of opioids to prevent coughing during emergence, which are higher in men than women [9,11]. In addition, enrolled patients in our study included both adult and geriatric groups. Yoo et al. demonstrated that remifentanil EC_50_ to suppress coughing after nasal surgery with sevoflurane anesthesia did not differ between elderly and adult patients [40]. However, cough, a defensive reflex of the respiratory tract, is impaired in elderly patients. Downregulation of the cough reflex in geriatric patients involves both cortical facilitatory pathways and medullary reflex pathways [41]. It is possible that age introduced bias in our findings. Further study is needed in patients with homogeneous sex and age to reduce these potential biases. Third, most patients who dropped out of the study showed delayed emergence. Administration of remifentanil may potentiate the hypnotic effect of anesthetics, which can cause delayed emergence from general anesthesia [42]. However, there were no statistical differences in recovery profile and remifentanil dose between the enrolled patient group and dropped out patient group. We could not find a clear reason for the relatively larger number of delayed emergence patients compared to other studies, but the unique characteristics of neurointervention including the minimal pain procedure and each patient’s individual susceptibility may be reasons.

## Conclusions

The EC_50_ and EC_95_ of remifentanil to prevent coughing for smooth emergence after endovascular neurointervention under sevoflurane anesthesia were 1.42 ng/mL and 1.70 ng/mL, respectively. Our results in this small sample indicate that the remifentanil concentration levels we utilized safely prevented coughing during emergence from sevoflurane anesthesia. The wide confidence intervals for our estimates (in particular, for EC95), however, indicate that a larger study would be useful to increase the precision of concentration levels.

## Supporting information

S1 File Research protocol of our study. (PDF)

S2 File Informed consent of our study (Korean). (PDF)

S3 File Informed consent of our study (English). (PDF)

S4 File TREND(Transparent Reporting of Evaluations with Nonrandomized Designs) statement checklist. (PDF)

## References

[1] Leech P, Barker J, Fitch W. Proceedings: Changes in intracranial pressure and systemic arterial pressure during the termination of anaesthesia. Br J Anaesth. 1974; 46:315–6. 10.1093/bja/46.4.315-a 4451611

[2] Saghaei M, Reisinejad A, Soltani H. Prophylactic versus therapeutic administration of intravenous lidocaine for suppression of post-extubation cough following cataract surgery: a randomized double blind placebo controlled clinical trial. Acta Anaesthesiol Taiwan. 2005; 43:205–9. 16450594

[3] Bidwai AV, Bidwai VA, Rogers CR, Stanley TH. Blood-pressure and pulse-rate responses to endotracheal extubation with and without prior injection of lidocaine. Anesthesiology. 1979; 51:171–3. 453622 10.1097/00000542-197908000-00020

[4] Lee JH, Choi SH, Choi YS, Lee B, Yang SJ, Lee JR. Does the type of anesthetic agent affect remifentanil effect-site concentration for preventing endotracheal tube-induced cough during anesthetic emergence? Comparison of propofol, sevoflurane, and desflurane. J Clin Anesth. 2014; 26:466–74. 10.1016/j.jclinane.2014.02.002 25200640

[5] Nasra J, Belvisi MG. Modulation of sensory nerve function and the cough reflex: understanding disease pathogenesis. Pharmacol Ther. 2009; 124:354–75. 10.1016/j.pharmthera.2009.09.006 19818366

[6] Choi SH, Min KT, Lee JR, Choi KW, Han KH, Kim EH, et al Determination of EC95 of remifentanil for smooth emergence from propofol anesthesia in patients undergoing transsphenoidal surgery. J Neurosurg Anesthesiol. 2015; 27:160–6. 10.1097/ANA.0000000000000094 25105828

[7] Lee SY, Yoo JY, Kim JY, Kim DH, Lee JD, Rho GU, et al Optimal effect-site concentration of remifentanil for preventing cough during removal of the double-lumen endotracheal tube from sevoflurane-remifentanil anesthesia: A prospective clinical trial. Medicine (Baltimore). 2016; 95:e3878 10.1097/MD.0000000000003878 27310976 PMC4998462

[8] Park YH, Chung EJ, Lee JH, Kim JT, Kim CS, Kim HS. Determination of the 95% effective dose of remifentanil for the prevention of coughing during extubation in children undergoing tonsillectomy (with or without adenoidectomy). Paediatr Anaesth. 2015; 25:567–72. 10.1111/pan.12616 25559991

[9] Dahan A, Kest B, Waxman AR, Sarton E. Sex-specific responses to opiates: animal and human studies. Anesth Analg. 2008; 107:83–95. 10.1213/ane.0b013e31816a66a4 18635471

[10] Hohlrieder M, Tiefenthaler W, Klaus H, Gabl M, Kavakebi P, Keller C, et al Effect of total intravenous anaesthesia and balanced anaesthesia on the frequency of coughing during emergence from the anaesthesia. Br J Anaesth. 2007; 99:587–91. 10.1093/bja/aem203 17660457

[11] Soh S, Park WK, Kang SW, Lee BR, Lee JR. Sex differences in remifentanil requirements for preventing cough during anesthetic emergence. Yonsei Med J. 2014; 55:807–14. 10.3349/ymj.2014.55.3.807 24719152 PMC3990090

[12] Joung KW, Yang KH, Shin WJ, Song MH, Ham K, Jung SC, et al Anesthetic consideration for neurointerventional procedures. Neurointervention. 2014; 9:72–7. 10.5469/neuroint.2014.9.2.72 25426301 PMC4239411

[13] Minto CF, Schnider TW, Egan TD, Youngs E, Lemmens HJ, Gambus PL, et al Influence of age and gender on the pharmacokinetics and pharmacodynamics of remifentanil. I. Model development. Anesthesiology. 1997; 86:10–23. 9009935 10.1097/00000542-199701000-00004

[14] Piccioni F, Mariani L, Bogno L, Rivetti I, Tramontano GT, Carbonara M, et al An acceleromyographic train-of-four ratio of 1.0 reliably excludes respiratory muscle weakness after major abdominal surgery: a randomized double-blind study. Can J Anaesth. 2014; 61:641–9. 10.1007/s12630-014-0160-7 24740312

[15] Dixon WJ. Staircase bioassay: the up-and-down method. Neurosci Biobehav Rev. 1991; 15:47–50. 2052197 10.1016/s0149-7634(05)80090-9

[16] Lee B, Lee JR, Na S. Targeting smooth emergence: the effect site concentration of remifentanil for preventing cough during emergence during propofol-remifentanil anaesthesia for thyroid surgery. Br J Anaesth. 2009; 102:775–8. 10.1093/bja/aep090 19411668

[17] Ashay NA, Wasim S, Anil TB. Propofol requirement for insertion of I-gel versus laryngeal mask airway: A comparative dose finding study using Dixon's up-and-down method. J Anaesthesiol Clin Pharmacol. 2015; 31:324–8. 10.4103/0970-9185.161666 26330709 PMC4541177

[18] Ezri T, Sessler D, Weisenberg M, Muzikant G, Protianov M, Mascha E, et al Association of ethnicity with the minimum alveolar concentration of sevoflurane. Anesthesiology. 2007; 107:9–14. 10.1097/01.anes.0000267534.31668.62 17585210

[19] Pace NL, Stylianou MP. Advances in and limitations of up-and-down methodology: a precis of clinical use, study design, and dose estimation in anesthesia research. Anesthesiology. 2007; 107:144–52. 10.1097/01.anes.0000267514.42592.2a 17585226

[20] Dilleen M, Heimann G, Hirsch I. Non-parametric estimators of a monotonic dose-response curve and bootstrap confidence intervals. Stat Med. 2003; 22:869–82. 10.1002/sim.1460 12627406

[21] Morris MD. Small-sample confidence limits for parameters under inequality constraints with application to quantal bioassay. Biometrics. 1988; 44:1083–92. 3233247

[22] Iasonos A, Ostrovnaya I. Estimating the dose-toxicity curve in completed phase I studies. Stat Med. 2011; 30:2117–29. 10.1002/sim.4206 21341302 PMC4849410

[23] Oron AP, Flournoy N. Centered Isotonic Regression: Point and Interval Estimation for Dose–Response Studies. Statistics in Biopharmaceutical Research. 2017; 9:258–67. 10.1080/19466315.2017.

[24] Kelly PD, Parker SL, Mendenhall SK, Bible JE, Sivasubramaniam P, Shau DN, et al Cost-effectiveness of cell saver in short-segment lumbar laminectomy and fusion (</ = 3 levels). Spine (Phila Pa 1976). 2015; 40:E978–85. 10.1097/BRS.0000000000000955 25929204

[25] Roberts GW, Bekker TB, Carlsen HH, Moffatt CH, Slattery PJ, McClure AF. Postoperative nausea and vomiting are strongly influenced by postoperative opioid use in a dose-related manner. Anesth Analg. 2005; 101:1343–8. 10.1213/01.ANE.0000180204.64588.EC 16243992

[26] Smith HS, Laufer A. Opioid induced nausea and vomiting. Eur J Pharmacol. 2014; 722:67–78. 10.1016/j.ejphar.2013.09.074 24157979

[27] Chang CH, Lee JW, Choi JR, Shim YH. Effect-site concentration of remifentanil to prevent cough after laryngomicrosurgery. Laryngoscope. 2013; 123:3105–9. 10.1002/lary.24199 23686891

[28] Guler G, Akin A, Tosun Z, Eskitascoglu E, Mizrak A, Boyaci A. Single-dose dexmedetomidine attenuates airway and circulatory reflexes during extubation. Acta Anaesthesiol Scand. 2005; 49:1088–91. 10.1111/j.1399-6576.2005.00780.x 16095449

[29] Neelakanta G, Miller J. Minimum alveolar concentration of isoflurane for tracheal extubation in deeply anesthetized children. Anesthesiology. 1994; 80:811–3. 8024135 10.1097/00000542-199404000-00013

[30] Minogue SC, Ralph J, Lampa MJ. Laryngotracheal topicalization with lidocaine before intubation decreases the incidence of coughing on emergence from general anesthesia. Anesth Analg. 2004; 99:1253–7, table of contents. 10.1213/01.ANE.0000132779.27085.52 15385385

[31] Fagan C, Frizelle HP, Laffey J, Hannon V, Carey M. The effects of intracuff lidocaine on endotracheal-tube-induced emergence phenomena after general anesthesia. Anesth Analg. 2000; 91:201–5. 10866913 10.1097/00000539-200007000-00038

[32] Mazzone SB, Undem BJ. Cough sensors. V. Pharmacological modulation of cough sensors. Handb Exp Pharmacol. 2009:99–127. 10.1007/978-3-540-79842-2_6 18825338

[33] Lee JH, Koo BN, Jeong JJ, Kim HS, Lee JR. Differential effects of lidocaine and remifentanil on response to the tracheal tube during emergence from general anaesthesia. Br J Anaesth. 2011; 106:410–5. 10.1093/bja/aeq396 21205628

[34] Molyneux A, Kerr R, Stratton I, Sandercock P, Clarke M, Shrimpton J, et al International Subarachnoid Aneurysm Trial (ISAT) of neurosurgical clipping versus endovascular coiling in 2143 patients with ruptured intracranial aneurysms: a randomised trial. Lancet. 2002; 360:1267–74. 12414200 10.1016/s0140-6736(02)11314-6

[35] P SL, Miskan MM, Y ZC, Zaki RA. Staggering the dose of sugammadex lowers risks for severe emergence cough: a randomized control trial. BMC Anesthesiol. 2017; 17:137 10.1186/s12871-017-0430-3 29020936 PMC5637258

[36] Doi M, Ikeda K. Airway irritation produced by volatile anaesthetics during brief inhalation: comparison of halothane, enflurane, isoflurane and sevoflurane. Can J Anaesth. 1993; 40:122–6. 10.1007/BF03011308 8443850

[37] Nishino T, Anderson JW, Sant'Ambrogio G. Effects of halothane, enflurane, and isoflurane on laryngeal receptors in dogs. Respir Physiol. 1993; 91:247–60. 8469848 10.1016/0034-5687(93)90103-h

[38] Ross AK, Davis PJ, Dear Gd GL, Ginsberg B, McGowan FX, Stiller RD, et al Pharmacokinetics of remifentanil in anesthetized pediatric patients undergoing elective surgery or diagnostic procedures. Anesth Analg. 2001; 93:1393–401, table of contents. 11726413 10.1097/00000539-200112000-00008

[39] Glass PS, Gan TJ, Howell S. A review of the pharmacokinetics and pharmacodynamics of remifentanil. Anesth Analg. 1999; 89:S7–14. 10511072 10.1097/00000539-199910001-00003

[40] Yoo JY, Kim JY, Kwak HJ, Lee DC, Kim GW, Lee SY, et al Effect-site concentration of remifentanil for preventing cough during emergence in elderly patients undergoing nasal surgery: a comparison with adult patients. Clin Interv Aging. 2016; 11:1247–52. 10.2147/CIA.S108705 27672319 PMC5026220

[41] Ebihara S, Ebihara T. Cough in the elderly: a novel strategy for preventing aspiration pneumonia. Pulm Pharmacol Ther. 2011; 24:318–23. 10.1016/j.pupt.2010.10.003 20937403

[42] Kern SE, Xie G, White JL, Egan TD. A response surface analysis of propofol-remifentanil pharmacodynamic interaction in volunteers. Anesthesiology. 2004; 100:1373–81. 15166554 10.1097/00000542-200406000-00007

