# Supplementary material for: Effect-site concentration of remifentanil for smooth emergence from sevoflurane anesthesia in patients undergoing endovascular neurointervention
Source: PLoS One. 2019 Jun 11;14(6):e0218074. doi: 10.1371/journal.pone.0218074 (PMC6559654; doi:10.1371/journal.pone.0218074)
Supplement: S1 File — (PDF) [file pone.0218074.s001.pdf]

Research Protocol

Project summary

In patients under endovascular neurointervention due to neurological disease, it is crucial to obtain gentle emergence from anesthesia without coughing since cough caused by endotracheal tube is associated with intracranial hypertension. Hemodynamic instability by stimulation can increase the incidence of intracranial bleeding or rupture of aneurysms. There have been efforts to prevent coughing while emerging from anesthesia and opioids are a commonly used therapeutic option. Previous studies demonstrated the antitussive effect of remifentanil for emergence from anesthesia under various settings and reported effective and safe suppression of coughing by target-controlled infusion (TCI) of remifentanil. However, various factors including patient age, sex, procedures, and anesthetic agents affect the Ce of remifentanil. Therefore, remifentanil administration to prevent coughing during emergence should be customized to the specific settings. The purpose of this study was to find the effective Ce of remifentanil to prevent coughing in 50% and 95% (EC_{50} and EC_{95}) of patients during emergence from sevoflurane anesthesia after endovascular neurointervention.

General information

- ✓ **Title: Effect-site concentration of remifentanil for smooth emergence from sevoflurane anesthesia in patients undergoing endovascular neurointervention**

- ✓ **Investigator**

  - Ji-Hye Kwon

- ✓ **Principal Investigator**

Ji Seon Jeong, M.D., Ph.D.

Tel.: +82-2-3410-2463; Fax: +82-2-3410-2461; E-mail: jiseon78.jeong@samsung.com

- ✓ **Institution**

Department of Anesthesiology and Pain Medicine, Samsung Medical Center, Sungkyunkwan University School of Medicine, Seoul, Korea

Rationale & background information

In patients under endovascular neurointervention due to neurological disease, it is crucial to obtain gentle emergence from anesthesia without coughing since cough caused by endotracheal tube is associated with intracranial hypertension. Hemodynamic instability by stimulation can increase the incidence of intracranial bleeding or rupture of aneurysms. Therefore, patients undergoing neurointervention should be gently recovered without coughing and hemodynamic instability. There have been efforts to prevent coughing while emerging from anesthesia and opioids are a commonly used therapeutic option to prevent and resolve coughing in patients with respiratory disease as well as with airway irritation. Previous studies demonstrated the antitussive effect of remifentanil for emergence from anesthesia under various settings and reported effective and safe suppression of coughing by target-controlled infusion (TCI) of remifentanil. However, various factors including patient age, sex, procedures, and anesthetic agents affect the Ce of remifentanil. Therefore, remifentanil administration to prevent coughing during emergence should be customized to the specific settings.

References (of literature cited in preceding sections)

1. Leech, P., Barker, J. & Fitch, W. Proceedings: Changes in intracranial pressure and systemic arterial pressure during the termination of anaesthesia. Br J Anaesth 46, 315-316 (1974).

Lee, J. H. et al. Does the type of anesthetic agent affect remifentanil effect-site concentration for preventing endotracheal tube-induced cough during anesthetic emergence? Comparison of propofol, sevoflurane, and desflurane. J Clin Anesth 26, 466-474 (2014).

Study goals and objectives

The purpose of this study was to find the effective Ce of remifentanil to prevent coughing in 50% and 95% (EC_{50} and EC_{95}) of patients during emergence from sevoflurane anesthesia after endovascular neurointervention.

Study Design

- ✓ **Design** : Prospective and observational study
- ✓ **Research population** : Adult patients aged from 20 to 70 years
- ✓ **Inclusion criteria**

  - ASA physical status of I-II and scheduled for neurointervention because of cerebrovascular disease

- ✓ **Exclusion criteria**

- Patients who had symptoms or signs of increased intracranial pressure,
- Malignant hypertension
- Preoperative administration of an antitussive agent
- Allergic history with use of remifentanil or contraindication to remifentanil
- Gastroesophageal reflux
- Arrythmia or congestive heart failure (New York heart association heart failure grade 3,4)
- Severe obesity (BMI > 35 kg/m2)
- Severe obstructive sleep apnea, anticipated difficult airway

- Acute or chronic respiratory disease (chronic obstructive lung disease, bronchial asthma, upper respiratory infection
- Patients who require ventilator care after neurointerventionon within 2 weeks, etc.)

✓ **Expected duration of study** : 1 year after IRB approval

Methodology

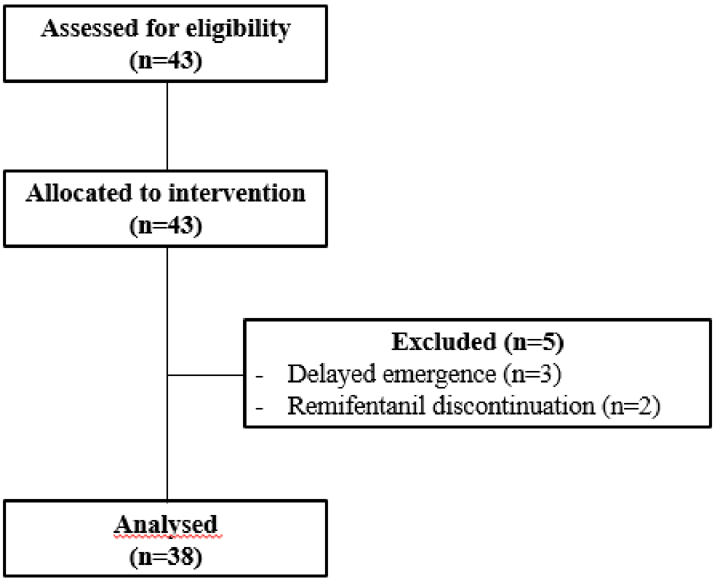

Figure 1. Consort diagram

✓ **Anesthetic protocol**

① Induction : Anesthesia was induced with 5 mg/kg of thiopental sodium and 4 vol% of sevoflurane and remifentanil effect site TCI of 2 ng/ml. For effect-site TCI of

remifentanil, a commercial TCI pump was used. After loss of consciousness, rocuronium 0.8 mg/kg was given intravenously, and manual ventilation with 100% O_2 was performed. Tracheal intubation was conducted.

② Monitoring : After intubation, supplementary monitors such as those for end-tidal carbon dioxide ($EtCO_2$), oroesophageal temperature, and invasive arterial blood pressure through a radial artery catheter were applied

③ Maintanence : Anesthesia was continued with sevoflurane and effect-site TCI of remifentanil to control a BIS target level of hypnosis of 40 to 60 and to control blood pressure and heart rate within 20% of baseline values.

④ About 15 minutes before procedure completion, sevoflurane concentration was controlled to maintain a BIS value of 60, and the Ce of remifentanil was set to a predetermined concentration.

⑤ After completion of the procedure, oropharyngeal suction was gently performed, and 2-4 mg/kg of sugammadex determined by TOF value was injected to reverse neuromuscular block. In addition, a predetermined concentration of remifentanil was maintained until extubation.

⑥ The patient received a verbal request continuously to open their eyes without any other stimulus. After eye opening, patients were asked to breathe deeply. When adequate spontaneous respiration was confirmed, ETT was removed with cuff deflated. Remifentanil was discontinued after extubation, and oxygen was supplied via facial mask for 3 minutes.

⑦ When the patient reached a stable state, she/he was moved to the postanesthetic care unit (PACU).

✓ **Determining remifentanil dose and blinding**

① Remifentanil was administered to prevent coughing during emergence using Dixon's up-and-down method.

② The initial Ce of remifentanil was 2.0 ng/ml for the first patient, based on the EC95 previously reported[6,7].

③ If the patient was determined to have a cough during emergence, it was considered as failure, and the next patient's Ce of remifentanil was increased by 0.2 ng/ml (10% of the initial Ce of remifentanil). Conversely, if the patient showed no cough during emergence, it was considered as success, and the next patient's Ce of remifentanil was decreased by 0.2 ng/mL.

✓ **Blinding**

Two anesthesiologists participated in the procedure. The first anesthesiologist titrated the remifentanil dose using a TCI pump and recorded remifentanil dose, end-tidal sevoflurane concentration (EtSevo), $EtCO_2$, and other hemodynamic parameters during the procedure and emergence. The second anesthesiologist who was blinded to the patients' Ce of remifentanil assessed the occurrence of cough and other complications during emergence and recorded the outcome. Recovery data including Richmond Agitation and Sedation Scale (RASS), modified Aldrete score, numeric rating scale (NRS), any complication, and duration of PACU stay were measured by the other investigator.

✓ **Outcomes**

- The EC_{50} and EC_{95} of remifentanil to prevent coughing during recovery
- The differences in intraoperative, emergence, and recovery data between the cough group and cough suppression group
-

✓ Evaluation

- Assessement of the occurrence of cough and other complications during emergence : Single investigator(JHK)
- Definition of cough : a strong and sudden contraction of the abdomen muscles during emergence

Safety Considerations

Moderate degree of adverse events associated with the study procedure (remifentanil administration) including hypotension, bradycardia. Acute respiratory depression

Severity of the adverse reaction is evaluated by the following criteria.

① Mild: It can be tolerated easily even though it has subjective or other symptoms.

② Moderate: It is uncomfortable enough to interfere with daily life.

③ Severe: To be able to perform normal daily life

In the all case of enrolled patients, the anesthesiologist specialist JHK and JSJ will perform close monitoring if the change is observed after surgery. If serious adverse events are

observed, they should be reported to the IRB and treated in accordance with in-hospital treatment standards.

During the study period, the researcher (anesthesiologist) performs vital monitoring and treats the problem if it is needed.

<Confidentiality of the subject> The record of the subject's identity will be kept confidential and the identity of the subject will remain confidential even when the results of the clinical trial are published. The subject's charts and case record information are kept confidential and kept in a confidential facility and in accordance with its management standards.

<Method of coding data> In all documents related to the clinical trial, such as case records, record and distinguish the patient's identification code (usually Case No.) rather than the patient's name.

Data Management and Statistical Analysis

- ✓ **Data** : All patient information is coded and documented.
- ✓ **Statistical analysis**
  - **The primary outcome** : Dixon's up-and-down method to determine EC_{50} and EC_{95} of remifentanil to prevent coughing
  - **The Secondary outcome :** Pearson correlation to evaluate correlations between Ce of remifentanil (total remifentanil dose) and time to extubation and duration of

PACU stay. Comparison of intraoperative, emergence, and recovery data between the cough suppression and cough group was performed using t-test, Wilcoxon rank sum test, chi-square test, and Fisher’s exact test.

Quality Assurance

1) Preservation of documents related to clinical trials: The institution conducting clinical trials shall preserve various data (including electronic documents) related to the conduct of clinical trials, including clinical trial plans and records on the management of clinical trial procedures, for a period of 10 years from the end of the study .

2) Confidentiality: Confidentiality of data and test subject records: All test subject identification information must be kept confidential, and the case record will include the test subject number and the test subject initials. The subject will be able to view the subject's medical records for the purpose of identifying the information collected by the IRB and the Food and Drug Administration and will inform that the information will be handled in a strictly confidential manner.

3) Clinical Trial Management Standards (KGCP, ICH E6): The procedures set out in this plan are based on the Clinical Trial Management Standards (KGCP: ICH E6) for testing,) And the fundamental spirit of the Helsinki Declaration.

4) Clinical trial review committee (IRB): Before starting the trial, the examiners should submit a copy of the clinical trial plan, the consent form, the data and procedures related to the recruitment of test subjects, It must be approved. The IRB's decision on the conduct of the test shall be communicated to the tester prior to commencement of the

test. The person responsible for the clinical trial will report to the IRB on the progress of the trial and any serious adverse events, life-threatening problems or missions, and notify the IRB at the end of the other tests.

5) Test subject's consent: The examiner should explain to the test subject (and / or his / her representative) participating in the clinical trial so that they can easily understand the nature of the test, the expected results, etc., , The test subject's written consent shall be signed and dated by the examiner and the subject and / or the agent. The original signed agreement must be kept by the tester and a copy must be given to the subject or the subject's representative. The examiner should not carry out any tests for the purpose of clinical studies before obtaining consent from the subject.

6) Surveillance Survey: The purpose of surveillance surveillance surveillance surveys is to ensure that all research activities and documents related to the research are carried out in accordance with the protocol, GCP, ICH guidelines and other regulatory requirements. And to examine them systematically and independently. The relevant authorities and the Food and Drug Administration may request that the relevant documents, case records, and other test documentation be viewed for inspection or surveillance, and the tester shall permit and cooperate with this process at all times. The researcher should immediately contact the relevant department of the regulatory agency if the investigation is scheduled.

Expected Outcomes of the Study

Patients undergoing neurointervention should be gently recovered without coughing and hemodynamic instability. Nonetheless, no specific reports on smooth emergence in patients going through endovascular neurointervention with sevoflurane anesthesia have been found.

Therefore, this study will provide useful clinical information about the optimal concentration of remifentanil to prevent coughing for patients undergoing neurointervention.

Dissemination of Results and Publication Policy

Corresponding author: Ji Seon Jeong, M.D., Ph.D

Duration of the Project

The study period is one year after the clinical trial approval. However, when the study is completed before that time, the time point is the end point of the study.

Project Management

Ji-hye Kwon : Patient collection, Data collection, Manuscript writing

Ji Seon Jeong : Study design, Manuscript writing, critical comments

Ethics

The investigator explained to the guardian of the patient one day before the anesthesia all protocols for this study for more than 30 minutes, and the informed consent was obtained after the patient's guardian understood it all

Informed Consent Forms

*Attached as additional files

Budget

This research received no specific grant from any funding agency in the public, commercial, or not-for-profit sectors.

Other support for the Project

None.

Collaboration with other scientists or research institutions

None.

Other research activities of the investigators

The Principal investigator (JSJ) contributed over 50% to study design and overall research participation in this study.

Financing and Insurance

Financing and insurance is based on the Samsung Medical Centre compensation standard.
