# Supplementary material for: Effect-site concentration of remifentanil for smooth emergence from sevoflurane anesthesia in patients undergoing endovascular neurointervention
Source: PLoS One. 2019 Jun 11;14(6):e0218074. doi: 10.1371/journal.pone.0218074 (PMC6559654; doi:10.1371/journal.pone.0218074)
Supplement: S2 File — (PDF) [file pone.0218074.s002.pdf]

연구 동의서

동의서 버전 또는 버전 날짜:	Version __1__ date __201 . . .__

연구 제목:	뇌혈관조영술을 받는 환자에서 안정적인 각성을 위한 remifentanil 의 EC95(95% 효과농도)와 EC50(50% 효과농도) 결정<br>Determination of EC95 of remifentanil for smooth emergence from sevoflurane anesthesia in patients undergoing trans-femoral cerebral angiography

연구책임자:	(성명)	정지선	(소속)	삼성서울병원	(연락처)	010-9153-8089
연구담당자:	(성명)	권지혜	(소속)	삼성서울병원	(연락처)	010-4917-2723
24 시간 연구자 연락처			(성명) 권지혜		(연락처)	010-4917-2723

*** 만일 본 연구에 문의사항이 있으시거나, 위험이나 불편 또는 손상이 발생할 경우, 상기 연구책임자 또는 연구담당자에게 연락하여 주시기 바랍니다.**

1. 참여 권유

본 연구책임자는 귀하로부터 임상시험 참여에 대한 동의를 받고 이를 문서화 할 때 관련 규정을 준수하며 헬싱키선언에 근거한 윤리적 원칙을 바탕으로 합법적인 절차를 따를 것입니다.

귀하는 본 임상시험에 참여할 것인지 여부를 결정하기 전에, 이 동의서를 신중하게 읽어보셔야 합니다. 이 연구가 왜 수행되며, 무엇을 수행하는지 귀하가 이해하는 것이 중요합니다. 이 임상시험에 대하여 설명한 아래 글을 읽으면서 어떤 질문이라도 할 수 있습니다. 충분한 시간을 가지고 결정해 주십시오.

귀하께서 궁금해 하는 모든 질문에 대해 답을 얻으셨고, 이 시험에 참여를 결정하였다면 본 동의서에 서명과 서명일자를 직접 적어야 합니다.

2. 본 임상시험은 연구 목적으로 수행됩니다.

3. 임상시험의 목적 및 배경

귀하가 시행하게 될 뇌혈관 조영술은 뇌혈관 질환의 진단과 치료에 필수적인 시술입니다. 연속촬영으로 얻은 영상으로 두개 내 혈류 상태와 측부순환의 상태를 파악하여 코일색전술과 같은 뇌혈관 질환 치료를 가능하게 합니다.

뇌혈관 질환으로 신경외과적 수술요법이나 신경중재적색전술 등의 치료를 받는 환자들에서 수술적 합병증을 예방하기 위해, 수술 혹은 시술 후 마취에서 깨어나는 과정에서 기침 없이 안정적으로 각성되는 것이 중요합니다. 각성과정에서 기관 삽관 튜브로 인해 상기도 자극되면서 기침이 발생하면, 고혈압, 빈맥, 두개내압 증가 등과 같은 부작용이 발생할 수 있기 때문입니다. 이러한 자극에 의한 급격한 혈역학적 변화는 시술부위 출혈 가능성을 높일 수 있으며, ICP 의 증가는 동맥류 파열이나 뇌혈관 손상 등으로 이어질 수 있습니다. 특히 귀하와 같이 뇌혈관 조영술을 받는 환자분들의 경우 뇌혈관 질환이 있거나 뇌현관중재술을 받은 환자들이기 때문에 수술 후 합병증 예방을 위해 안정적인 각성이 필수적입니다. 하지만 지금까지 TFCA 를 받는 환자에서 안정적 각성에 대한 연구는 부족한 실정입니다.

현재 본원에서는 기침의 억제 및 혈얍조절을 위하여 레미펜타닐을 사용하고 있습니다. 레미펜타닐은 전신마취를 시행할 때 통증과 혈압 조절을 위하여 병용투여되는 약물로서, 진해 효과를 가지고 있으며, 마취의 각성과정에서 효과적으로 기침을 억제하는 것으로 알려져 있습니다. 따라서 본 연구에서는 뇌혈관조영술을 받는 95%(50%)의 환자들이 세보플루레인 마취에서 기침 없이 안정적으로 각성할 수 있는 레미펜타닐의 효과농도를 측정하고자 합니다.

4. 임상시험에 사용되는 두 그룹에 무작위로 배정될 확률

본 임상시험에서 사용되는 약제는 레미펜타닐 입니다. 상기 언급한 약제는 이미 임상적으로 검증되어 상용화 중인 방법으로, 본 임상시험에서는 기침을 억제할 수 있는 약물의 용량을 알아 보고자 합니다. 귀하가 시험 참가에 동의하시면 귀하에게 투여되는 약물의 용량은 이전 시험에 따르거나 우연에 의해(동전 던지기와 같이) 결정됩니다.

5. 임상시험 참여에서 종료 시까지 검사 및 절차

본 임상시험에서 귀하에게는 이전 연구 결과에 따라 정해진 용량의 약물이 투여될 것입니다. 귀하나 귀하의 기침 여부를 평가하는 의사는 귀하에게 투여된 약물용량을 알 수 없습니다.

귀하의 본 임상시험 참여는 시술을 받은 당일 및 수술 후 3 시간에 해당됩니다.

먼저, 시험 담당 의사가 이번 연구의 목적을 설명합니다. 귀하가 이번 시험에 참여하기로 결정하는 경우, 귀하는 이 동의서를 읽고 서명할 것을 요청 받습니다.

귀하는 전신마취 하에 뇌혈관조영술을 받게 됩니다. 전신마취 시행 중에는 기계호흡을 실시하게 되며, 이를 위해 기관 내 삽관 및 근이완제를 투여하게 됩니다. 시술 중 마취 심도를 감시하기 위하여 이마에 BIS 를 붙이게 됩니다. 시술 중에는 지속적으로 혈압, 맥박, 산소포화도, 심전도, 마취심도 등을 감시합니다. 만약 시술 도중 귀하의 마취 심도나 생체 징후에 변화가 생길 경우, 본 의료진은 귀하의 안전을 위하여 약물을 투여하여 조절 하도록 할 것입니다.

시술이 종료된 뒤 마취가스를 중지하고, 근이완의 역전을 위하여 수가마덱스를 투여하게 됩니다. 이전 실험에 따라 정해진 농도의 레미펜타닐을 기관발관 시까지 유지합니다. 귀하는 눈을 뜨라는 언어 자극 외에 다른 자극을 받지 않고 자발 호흡이 완전히 돌아왔음이 확인되면 기관발관이 시행됩니다. 기간발관 직 후 레미펜타닐을 중단하고 5 분간 산소마스크로 산소를 공급한 뒤 환자는 회복실로 이송합니다. 회복실 이동 후 1 시간 가량 생체징후 및 진정정도, 부작용 발생여부등을 감시하게 됩니다.

본 연구진은 시술 후 회복실 체류 기간동안 귀하가 경험할 수 있는 부작용 및 합병증 여부를 관찰 및 기록하고, 필요시 의료진의 판단에 따라 적절한 치료가 시행될 것입니다.

임상시험은 시술 후 회복실 퇴실(대략 1-3 시간) 후 종료하게 됩니다.

6. 본 시험을 위해서 귀하가 준수해야 하는 사항

본 임상시험은 귀하가 시술을 받는 도중 및 시술 후의 혈역학적 징후 및 기침유무를 관찰하는 시험으로, 귀하가 준수해야 하는 사항은 없습니다.

7. 본 임상시험의 검증되지 않은 실험적인 측면

본 연구에서는 뇌혈관조영술을 받는 환자에서의 기침 억제를 위한 레미펜타닐의 용량을 알아보는 실험적인 측면이 검증되지 않았습니다.

8. 임상시험 참여로 인하여 예견되는 위험(부작용)이나 불편사항

본 연구에 참여하는 동안, 귀하는 아래에 나열된 알려진 부작용과 그밖에 예측하지 못한 부작용을 경험할 위험이 있습니다. 귀하의 연구담당 의사가 이들에 대해 귀하에게 상의드릴 것입니다. 부작용은 사람에 따라 다양하며, 귀하는 이들 부작용을 모두 또는 일부를 경험하거나 전혀 경험하지 않을 수도 있습니다. 귀하가 부작용을 경험한다면, 귀하의 연구담당 의사에게 알려서 귀하의 고통을 치료하거나 귀하가 경험한 불편을 해소하기 위한 방법에 대해 안내 받으실 수 있습니다.

- 레미펜타닐 관련 부작용 : 급성호흡기억제, 저혈압, 서맥, 골격근의 강직 등의 μ-아편양 작용제의 약리작용의 직접적인 확대

9. 본 임상시험에 참여함으로써 기대되는 이익

귀하가 이 임상시험에 참여하더라도 귀하에게 직접적으로 기대되는 이익은 없습니다. 그러나 이 연구를 통해 뇌혈관조영술을 받는 환자에서의 기침 억제를 위한 레미펜타닐의 용량을 알게 된다면 향후 귀하가 같은 시술을 받게 되거나 같은 시술을 받는 다른 환자에게 도움을 줄 수 있습니다.

10. 본 질환으로 선택할 수 있는 다른 치료방법 및 이러한 치료의 잠재적 위험과 이익

귀하는 본 임상시험에 반드시 참여해야만 하는 것은 아닙니다. 본 임상시험에 참여하지 않는다 하여도 귀하의 마취 중 혈압 조절 및 기침억제를 위하여 위하여 시험약제가 사용되어야 합니다. 시험약제가 사용되지 않을 경우 진해 효과를 위한 다른 약제 (벤프로페린 등)을 사용할 수 있으나 본원에서는 마취중 사용하지 않는 약물이며, 레미펜타닐이 만큼 즉각적인 효과를 기대할 수 없어 이에 따른 기침발생으로 인한 부작용 발생의 위험성이 있습니다.

11. 예상 참여기간 및 본 시험에 참여하는 대략의 전체 연구대상자 수

본 임상시험에 참여하게 되면 시술을 받고, 시술 후 3 시간이내로 추적관찰을 받게 됩니다. 본 연구에는 본원 환자 20 - 40 명이 참여할 예정입니다.

12. 임상시험과 관련된 손상이 발생하였을 경우의 보상/배상이나 치료방법

본 연구에서는 이미 치료제로 사용되고 있는 약물을 투약하는 연구로, 연구에 참여에 따른 손상에 대해 추가적으로 제공되는 보상은 없습니다. 약물 투여에 따른 부작용 발생의 경우는 이미 알려진 치료방법으로 치료 받게 됩니다.

13. 임상시험에 참여함으로써 받게 되는 금전적 보상의 여부 및 추가적으로 발생이 예상되는 비용

본 임상시험에 참여함으로써 제공되는 금전적인 보상도 없습니다. 본 임상시험에 사용하게 되는 약물(레미펜타닐)은 이미 임상에서 표준치료제로 사용되고 있는 의약품이므로 의약품에 대한 비용은 본인이 부담하셔야 합니다. 의약품 비용 이외의 추가적인 검사에 시행되는 비용은 없습니다.

14. 연구참여의 제한

다음에 해당되는 경우, 귀하는 귀하의 동의 없이도 본 연구의 참여로부터 제한될 수 있습니다.

A. 시험 담당의사의 지시를 따르지 않음

B. 시험 담당의사가 귀하에게 있어 연구가 최선의 방법이 아니라고 결정함

15. 임상시험 지속 참여 의지에 영향을 줄 수 있는 새로운 정보

이 연구에 대한 참여는 전적으로 귀하의 선택입니다. 이 연구에 참여할 귀하의 의지에 영향을 줄 수 있는 새로운 정보가 수집되면 제때에 귀하 또는 대리인에게 알려드릴 것입니다.

16. 자유의사에 의한 시험 참여 동의 및 철회 및 시험중단 이후의 절차

본 임상시험 참여는 전적으로 자발적입니다. 임상시험 참여 여부를 결정하는 것은 귀하에게 달려있습니다. 만약 귀하가 시험 참여를 결정하였더라도 귀하는 언제든지 이유를 대지 않고 임상시험 참여를 자유롭게 중단할 수 있습니다. 만약 귀하가 임상시험에 불참 또는 참여 중단을 선택하였더라도 이것이 향후 귀하의 의학적 치료에 어떠한 방식으로도 영향을 미치지 않을 것입니다.

또한 참여에 동의한 이후에도 동의 철회를 원할 경우 조사된 자료를 폐기하도록 요청하실 수 있으며 이 경우 이미 연구에 사용된 정보와 자원을 제외하고 모든 자료는 적법한 절차에 따라 폐기됩니다.

17. 개인정보 보호

만약 귀하가 본 연구에 참여하신다면 본 연구에서는 귀하의 개인정보(성명과 같은 개인 식별정보 및 건강에 관한 정보 등)를 수집하게 됩니다. 이렇게 수집된 개인정보는 관련 법규에 따라 엄격하게 관리되며 연구에 관련된 담당자만이 수집된 자료에 접근할 수 있습니다. 수집된 개인정보 중 개인식별정보는 연구에 직접 이용되거나 필요한 정보가 아니며 임상 자료와 귀하를 연결하기 위한 목적으로만 사용됩니다. 귀하의 개인정보는 연구 목적을 달성할 때까지 사용하게 되며 수집된 정보는 개인정보보호법에 따라 적절히 관리됩니다.

귀하의 신상을 파악할 수 있는 기록은 비밀로 보호될 것이며 임상시험의 결과가 출판될 경우에도 귀하의 신상은 비밀로 보호될 것입니다. 다만, 모니터요원, 점검을 실시하는 자, 심사위원회(IRB) 및 식품의약품안전처장이 관계 법령에 따라 임상시험의 실시절차와 자료의 품질을 검증하기 위하여 연구대상자의 신상에 관한 비밀이 보호되는 범위에서 연구대상자의 의무기록을 열람할 수 있지만 이 경우에도 최대한 비밀이 보호되도록 할 것입니다. 귀하(또는 대리인)가 서명한 동의서에 의하여 이러한 자료의 열람이 허용됩니다. 임상시험과 함께 귀하의 전자차트의 기록을 review 하게 되므로, 귀하의 의료정보 중 신원을 직접적으로 파악할 수 있는 정보는 신뢰할 수 있는 방법으로 보호하며 사용하도록 할 것 입니다. 의무기록 열람에 있어 식별할 수 있는 이름은 이니셜로 기록하고, 병원 등록 번호 대신 식별코드를 부여하며 식별코드지는 암호화하여 파일로 보관할 예정입니다.

18. 연구대상자로서의 권익에 관한 정보 제공

본 임상시험은 본원 연구대상자의 권리, 안전, 복지를 보호할 책임이 있는 기관윤리심의위원회(IRB)에 의해 승인되었으며 본 연구의 대상자로서 귀하의 권리에 대해 질문이 있으시면 당원에서 지정한 "피험자보호 연구윤리 담당자(TEL 02-3410-2980)"에게 문의하실 수 있습니다. 본 시험에 참가하기로 선택하였다면 귀하는 서명된 동의서의 사본을 받게 될 것입니다.

19. 연구대상자 동의

✓ 본인은 본 동의서의 내용에 대해 충분히 설명을 들었고 시험 담당의사 또는

연구진과 함께 해당 내용에 대해 상의했습니다.

- ✓ 본인은 동의서 내용을 읽고 이해하였으며 질문할 기회를 가졌고 본인의 모든 질문에 대해 만족스러운 답변을 받았습니다.
- ✓ 본인은 자발적 의사로 본 임상시험에 참여하는 것에 동의합니다. 본 동의서에 서명하더라도 본인은 본인의 권리를 포기하지 않습니다
- ✓ 본인은 언제든지 자유롭게 본 임상시험에 대한 동의를 철회할 수 있으며 또한 이로 인하여 본인의 진료나 권리에 영향이 발생하지 않을 것임을 알고 있습니다.
- ✓ 본인은 동의 후에 서명 및 날짜가 기재된 설명문 및 동의서의 사본을 제공받을 것임을 알고 있습니다.

연구대상자 성명: ______________________

서명: ______________________ 날짜: ______________________

연구대상자 대리인: ______________________ 대상자와의 관계: ______________
(필요한 경우)
서명: ______________________ 날짜: ______________________

연구책임자/공동연구자 성명: ______________

서명: ______________________ 날짜: ______________________

연구대상자가 본인이 읽을 수 없다는 의사를 표현한 경우로, 연구자가 본 동의서를 연구대상자 또는 대리인에게 읽어 주었고 연구대상자와 함께 이를 논의하였으며 질문할 기회를 제공함을 확인합니다

참관인 성명: ______________________ 비고(관계, 신분, 입회사유 등): __________
(글을 읽지 못하는 연구대상자의 경우)
서명: ______________________ 날짜: ______________________

.

본 동의서는 삼성서울병원 기관윤리심의위원회(IRB)에서 심의하여
사용을 승인한 동의서로. SMC 철인이 된 경우에만 유효합니다
