# Supplementary material for: Effect-site concentration of remifentanil for smooth emergence from sevoflurane anesthesia in patients undergoing endovascular neurointervention
Source: PLoS One. 2019 Jun 11;14(6):e0218074. doi: 10.1371/journal.pone.0218074 (PMC6559654; doi:10.1371/journal.pone.0218074)
Supplement: S3 File — (PDF) [file pone.0218074.s003.pdf]

Informed Consent Form

Version	**Version __1__ date __201 . . .__**
Name of research:	Determination of EC95 of remifentanil for smooth emergence from sevoflurane anesthesia in patients undergoing trans-femoral cerebral angiography

Principal Investigator:	(Name)	Ji Seon Jeong	(Organization)	Samsung medical center	(Contact)	010-9153-8089
Study doctor:	(Name)	Ji-Hye Kwon	(Organization)	Samsung medical center	(Contact)	010-4917-2723
24 hour Contact		(Name) Ji-Hye Kwon			(Contact)	010-4917-2723

***** If you have any questions about this study, or if there is any danger, inconvenience or damage, please contact the research director or researcher..

1. Invitation to participate

Your supervisor will agree to participate in clinical trials and will comply with the relevant regulations when you document them and will follow legal procedures based on ethical principles based on Helsinki.

You must read this agreement carefully before deciding whether or not you will participate in the trial. It is important that you understand why this study is carried out and what it does. As you read the following article on this trial, you can ask any questions. Please have enough time to decide.

If you have answered all the questions that you are wondering and you have decided to participate in this exam, you must sign this agreement and sign it yourself.

2. This clinical trial is conducted for research purposes.

3. Purpose and Background of Clinical Trial

The objective of the study is to estimate the effective Ce of remifentanil for smooth emergence without coughing from sevoflurane anesthesia in 95% and 50% of subjects undergoing Trans-femoral cerebral angiography for intervention.

Cerebral angiography is a form of angiography which provides images of blood vessels in and around the brain, thereby allowing detection of abnormalities such as arteriovenous malformations and aneurysms. For some applications, cerebral angiography may yield better images than less invasive methods such as computed tomography angiography and magnetic resonance angiography. In addition, cerebral angiography allows certain treatments to be performed immediately, based on its findings. If, for example, the images reveal an aneurysm, metal coils may be introduced through the catheter already in place and maneuvered to the site of aneurysm.

In patients under Trans-femoral cerebral angiography due to neurologic disease, it is important to achieve smooth emergence from anesthesia without coughing because coughing against the endotracheal tube is associated with adverse events such as hypertension, tachycardia, and intracranial hypertension. Trans-femoral cerebral angiography requires smooth emergence from anesthesia to reduce the incidence of above mentioned complications. Hemodynamic stimulation may increase the chance of intracranial bleeding or rupture of aneurysm s. Therefore, the patients under Trans-femoral cerebral angiography should recover smoothly without coughing and hemodynamic instability. However, to our best knowledge, there is no specific study on smooth emergence in patients undergoing Trans-femoral cerebral angiography.

In recent studies, the antitussive effect of remifentanil during recovery from general anesthesia was reported by demonstrating that a target-controlled infusion (TCI) of remifentanil using a predicted effect-site concentration (Ce) can safely and effectively suppress coughing induced by an endotracheal tube. The objective of the study was to estimate the effective Ce of remifentanil for smooth emergence without coughing from sevoflurane anesthesia in 95% (50%) of subjects undergoing Trans-femoral cerebral angiography.

4. The probability of being randomly assigned to the two groups used in clinical trials

The drug used in this trial is remifentanil. The above-mentioned drugs have already been clinically proven and are being commercialized. In this trial, we would like to know the dosage of drugs that can inhibit cough. If you agree to participate in the trial, the dose of the drug administered to you will be reduced by 0.2 ng / mL if the cough is effectively inhibited from previous test results at an initial dose of 2.0 ng / mL, Drugs will be dosed according to the subject's condition within the approved dose.

5. Testing and procedures from participation in clinical trials to completion

In this trial, you will be given a dose of drug based on the results of previous studies. Two doctors are involved in anesthesia, the first doctor will know and control the amount of remifentanil, and the second doctor will assess your cough without knowing the dosage.

Your participation in this trial will be on the day of surgery and 3 hours after surgery.

First, the exam taker explains the purpose of this study. If you decide to participate in this exam, you will be asked to read and sign this agreement. You will undergo cerebral angiography under general anesthesia. During general anesthesia, mechanical ventilation is performed. To do this, intubation and muscle relaxation are performed. To monitor the depth of anesthesia during the procedure, a BIS (bison spectral index; a monitoring device that measures the calmness of the anesthesia patient) is placed on the forehead. During the procedure, blood pressure, pulse, oxygen saturation, electrocardiogram, and anesthetic depth are measured continuously. If there is a change in your anesthetic depth or vital signs during the procedure, you will be given medication for your safety.

After the procedure is completed, the anesthetic gas is stopped and Su Gamma Dex is administered to reverse the muscle relaxation. According to the previous experiment, a fixed concentration of remifentanil is maintained until the tracheal tube (removing the tubes that are intubated in the airways). If you notice that the spontaneous breathing has returned completely without any stimulation other than the language stimulus that opens your eyes, the ventilator will be performed. Immediately after the ventilation, the remifentanil is stopped, oxygen is supplied by the oxygen mask for 5 minutes, and the patient is transferred to the recovery room. We will monitor the vital signs, calmness, and side effects for about an hour after the recovery room is moved.

We will observe and record any side effects and complications you may experience during your stay in the recovery room after the procedure, and if necessary, appropriate treatment will be performed at the discretion of the medical staff.

Clinical trials will end after leaving the recovery room (approximately 1-3 hours) after the procedure.

6. What you must observe for this exam

This is a test to observe hemodynamic signs and coughs during and after your procedure. There is nothing you need to observe.

7. Untested experimental side of the trial

This study did not validate the experimental aspect of remifentanil dosing for cough suppression in patients undergoing angiography.

8. Risk (side effects) or discomfort predicted by participation in clinical trials

During your participation in this study, you are at risk of experiencing the known side effects listed below and any other unforeseen side effects. Your research doctor will talk to you about these. Side effects vary from person to person, and you may or may not experience all or part of these side effects. If you experience side effects, you can tell your research doctor about how to cure your pain or resolve any discomfort you may have experienced.

- Remifentanil Side Effects

: Acute respiratory distress, Hypotension, Bradycardia, Direct enlargement of the pharmacological action of μ-opioid agonists such as skeletal muschle stiffness

9. Expected benefits from participation in this trial

Even if you participate in this trial, there is no immediate benefit to you. However, if this study reveals the dose of Remifentanil for cough suppression in patients undergoing angiography, you may help another patient who receives the same treatment.

10. Other treatment options that can be selected for this disease and the potential risks and benefits of such treatment

You are not required to participate in this trial. Even if you do not participate in this trial, remifentanil (test medication) should be used to control blood pressure and cough suppression during general anesthesia. If the test agent is not used, other agents (such as benproperin) may be used for the calming effect but this is a drug not used during anesthesia in our hospital. Moreover, this is not as effective as remifentanil so there is a risk of side effects from coughing.

11. Anticipated duration of participation and approximate total number of subjects participating in the trial

When you participate in this clinical trial, you will receive the procedure and follow up within 3 hours after the procedure. In this study, 20-40 patients will be participating in this study.

12. Compensation or treatment in the event of a clinical trial-related injury

In this study, a drug that has already been used as a therapeutic agent, there is no additional compensation for damage caused by participation in the study. In the event of adverse drug reactions, you will be treated with a known treatment.

13. The amount of monetary compensation you will receive from participating in clinical trials and the expected additional costs

There are no monetary rewards from participating in this trial. Since the drug (Remifentanil) used in this clinical trial is a drug already used as a standard treatment in clinical practice, the cost of medicine should be paid by the person himself. There is no cost for additional inspections other than drug costs.

14. Limitations of participation in research

If you meet the following conditions, You may be restricted from participation in this study without your consent.

A. *Your test doctor has decided that research is not the best way for you*

15. New information that may influence the commitment to continue clinical trials

Participation in this study is entirely your choice. We will notify you or your guardians on time as new information is collected that may affect your willingness to participate in this study.

16. Procedures after consent to withdrawal and withdrawal from the trial by a free doctor

Participation in this trial is entirely voluntary. It is up to you to decide whether or not to participate in the clinical trial. Even if you decide to participate in the trial, you are free to stop participating in the trial at any time without reason. If you choose not to participate in the clinical trial or choose to stop participating, this will not affect your medical treatment in any way in the future.

In addition, if you wish to withdraw your consent after your consent to participation, you may be asked to dispose of the investigated material, in which case all material will be destroyed in accordance with the legitimate procedure, except for the information and resources already used in the study.

17. Privacy

If you participate in this study, we will collect your personal information (such as personally identifiable information and health information such as your name). The collected personal information is strictly controlled in accordance with the relevant laws and regulations, and only the personnel involved in the research can access the collected data. Of the personal information collected, personally identifiable information is not used directly or necessary information in the research and is used only for the purpose of linking you with the clinical data. Your personal information will be used until the research purpose is achieved, and the information collected will be appropriately managed according to the privacy laws.

Records that can identify your personal secrets will be protected by Your personal even if the results of the clinical trials to be published will be protected as confidential. However, monitoring personnel, who conducted the inspection, examination Board (IRB) and the Food and Drug Dean of the laws extent that the secret of the statue of subjects protection in order to verify the implementation process and data quality in clinical trials, according to You can view the subject's medical records in this case, but we will try to keep the secret as much as possible. You are permitted to view these materials by a written agreement signed by you (or your guardians). Since the review of the records of your electronic chart with clinical trials, will be information that can directly identify the identity of your medical information will be used and protected in a reliable way. In reading medical records that can be identified by initials, names written, and given the registration number and identification code instead of the hospital will keep a file that is encrypted identification code.

18. Providing information on rights and interests of research subjects

This study was approved by the Institutional Ethics Review Board (IRB), which is responsible for protecting the rights, safety and welfare of the subjects of this study. If you have any questions about your rights as a subject of this study, You can contact the Ethics Officer (TEL 02-3410-2980). If you choose to participate in this exam, you will receive a copy of the signed agreement.

19. Consent of research subjects

- ✓ I have been fully informed of the content of this agreement and have discussed it with my test taker or research team.
- ✓ I have read, understood and have the opportunity to ask questions and have received satisfactory answers to all my questions.
- ✓ I voluntarily agree to participate in this clinical trial. Even if I sign this agreement, I will not give up my rights.
- ✓ I understand that I may freely withdraw my consent to this trial at any time and that this will not affect my treatment or my rights.
- ✓ I understand that I will be given a signed and dated copy of the written explanation and consent form after my consent.

Research Subject Name: ____________

Signature: ____________ Date: ____________

Guardians of subject: ____________ Relationship: ____________
(If needed)
Signature: ____________ Date: ____________

Principal Investigator/Co-researcher Name:____

Signature: ____________ Date: ____________

.

This agreement is a written consent approved by the IRB of the Samsung Medical Center.
